# Makerspace network embeddedness, business model innovation, and user entrepreneurial performance in China: The moderating effect of environmental dynamics

**DOI:** 10.1371/journal.pone.0322388

**Published:** 2025-04-29

**Authors:** Jinbo Zhou, Weiren Cen, Yuzhi Ling

**Affiliations:** School of Economics and Management, Guangxi Normal University, Guilin, China.; West Pomeranian University of Technology, POLAND

## Abstract

Makerspaces gather a large number of entrepreneurial entities and resources, playing a vital role in the survival and development of user entrepreneurial enterprises. Based on network embeddedness theory and resource-based theory, this study employs hierarchical regression analysis and bootstrap method to examine the relationship between makerspace network embeddedness, business model innovation, and user entrepreneurial performance, as well as the moderating role of environmental dynamics. Using empirical data from 245 valid samples of user entrepreneurial firms in Chinese makerspaces, the results reveal that makerspace network embeddedness significantly promotes business model innovation and user entrepreneurial performance. Moreover, business model innovation has a significantly positive impact on user entrepreneurial performance, and partially mediates the relationship between makerspace network embeddedness and user entrepreneurial performance. Furthermore, environmental dynamics exhibit a significant positive moderating effect on the relationship between makerspace network embeddedness and business model innovation. These findings effectively reveal the mechanism for enhancing user entrepreneurial performance and offer practical insights for user entrepreneurial enterprises to leverage network embeddedness for improved entrepreneurial performance.

## 1. Introduction

Under the deepening implementation of China’s Mass Entrepreneurship and Innovation strategy, innovation and entrepreneurship have been widely recognized as key drivers of high-quality economic development [1]. The successful emergence of user-led entrepreneurial ventures such as Unitree Robotics, DeepSeek, and Bilibili marks the rapid development of a new entrepreneurial paradigm dominated by user entrepreneurs [2]. User entrepreneurs are typically intensive users of products who innovate to develop solutions in response to unmet needs in existing products or services [3]. After validating the feasibility and market potential, they proactively engage in business model innovation within user communities [2]. However, unlike other types of entrepreneurs, they commonly face three persistent challenges during commercialization: insufficient resource acquisition and integration capabilities, high external environmental uncertainty, and low maturity of business models, necessitating empowerment from external support systems [4]. As a strategic solution to these challenges, makerspaces are shared physical or virtual environments designed to provide entrepreneurs with essential innovation resources, including prototyping tools, technical support, mentorship, funding channels, and collaborative networks. By constructing open innovation platforms and integrating diverse entrepreneurial actors and resources, makerspaces foster the formation of a highly interactive and collaborative network embeddedness ecosystem, reducing innovation barriers and accelerating the commercialization of innovative outcomes [2,5]. The concept of network embeddedness, rooted in Granovetter’s embeddedness theory, refers to the process by which firms or individuals establish connections with external entities through specific social network structures to acquire resources, information, and opportunities [6,7]. In this context, a higher degree of network embeddedness can enhance resource mobilization, promote knowledge exchange, and improve market adaptability, thereby accelerating the commercialization of user-driven innovations.

From an integrated perspective of resource-based theory and network embeddedness theory, firms must achieve competitive advantage through business model innovation, which fundamentally involves the redesign or optimization of value creation, delivery, and capture mechanisms [7–9]. Structural embeddedness and relational embeddedness serve as critical pathways for firms to establish external linkages, enabling efficient acquisition of scarce resources and driving business model innovation [9–11]. The synergy of these theoretical lenses highlights the dual mechanisms through which makerspaces operate: structural embeddedness allows user ventures to access the holistic network architecture of makerspaces, securing critical resources such as technology, capital, and talent to compensate for resource deficiencies [11–13]; relational embeddedness fosters deep trust-based relationships with investors, suppliers, and partners, forming strategic alliance networks that reduce transaction costs and enhance knowledge spillovers and collaborative innovation [13–15]. Collectively, this dual embeddedness mechanism not only alleviates resource constraints but also strengthens innovation capabilities and competitive advantages through social capital accumulation and network effects, thereby overcoming early-stage bottlenecks and catalyzing business model innovation to improve entrepreneurial performance [15–18].

Resource-based theory emphasizes that environmental dynamics play a pivotal moderating role in the process of heterogeneous resource acquisition and business model innovation [19]. External factors such as competitive landscapes, policy support, and industry regulations profoundly influence firms’ capacity for business model innovation and, consequently, user entrepreneurial performance [20]. In the context of rapid technological iteration and industrial transformation, user ventures face challenges like intensified competition and volatile demand while simultaneously benefiting from policy incentives and technological advancements [21–22]. This dynamic environment may amplify the resource acquisition effects of network embeddedness while also reshaping the efficacy of business model innovation [23]. Thus, investigating the moderating role of environmental dynamics in the makerspace network embeddedness–business model innovation–user entrepreneurial performance chain holds significant theoretical value for understanding the growth mechanisms of user ventures [24]. Integrating resource-based theory and network embeddedness theory, this study employs hierarchical regression and bootstrap methods to address three research questions:

RQ1: How does makerspace network embeddedness influence user entrepreneurial performance through dual mechanisms of structural and relational embeddedness?
RQ2: What mediating role does business model innovation play between makerspace network embeddedness and user entrepreneurial performance?
RQ3: How does environmental dynamics moderate the impact of makerspace network embeddedness on business model innovation and the subsequent effect of innovation on entrepreneurial performance?

The structure of this paper is organized as follows. First, a comprehensive literature review is conducted on the themes of makerspace network embeddedness, business model innovation, and user entrepreneurial performance, based on which research hypotheses are proposed. Second, the research methodology is elaborated in detail, including the data collection process and the measurement of variables. Third, the empirical results are presented, highlighting the key findings derived from the analysis. Fourth, the findings are discussed in relation to their alignment, divergence, or complementarity with existing studies. Finally, the conclusions are summarized, the theoretical and practical implications of the research are discussed, and future research directions are proposed.

## 2. Literature review and hypotheses development

### 2.1. Makerspace network embeddedness and user entrepreneurial performance

User entrepreneurial performance, a critical indicator of entrepreneurial success, encompasses multiple dimensions, including survival performance, growth performance, innovation performance, and financing performance [25–26]. It not only comprehensively reflects the market competitiveness, innovation capability, and resource acquisition ability of user entrepreneurial ventures but also provides real-time feedback to entrepreneurs, enabling them to identify and address issues promptly [27]. Grounded in network embeddedness theory, the economic activities of firms are embedded within social network structures and are influenced by interactions with other network members, such as organizations, governments, and communities [28]. This embeddedness allows user entrepreneurial ventures to expand resource acquisition channels and enhance resource utilization efficiency through interactions with external networks, thereby exerting a profound impact on entrepreneurial performance [29].

Under the combined framework of network embeddedness theory and resource-based theory, the mechanisms of resource acquisition and competitive advantage are clearly articulated [30]. Granovetter categorizes network embeddedness into two dimensions: structural embeddedness and relational embeddedness [31]. Structural embeddedness reflects a firm’s position and connectivity within a social network through metrics such as network centrality and network density [32]. Higher makerspace network density provides user entrepreneurial ventures with richer resource channels, more interaction opportunities, and lower resource acquisition costs, thereby supporting the construction of competitive advantages [33]. Relational embeddedness, on the other hand, emphasizes trust, interaction frequency, and the durability of connections among members [34]. Higher relational embeddedness strengthens trust among members, facilitates smoother information and resource flows, and enhances the efficiency of sharing and utilizing scarce resources [35]. From the perspective of resource-based theory, a firm’s competitive advantage stems from its unique and scarce resources [36]. Relational embeddedness within makerspaces enhances trust, providing firms with deeper resource acquisition pathways and enabling them to efficiently integrate external resources, thereby driving entrepreneurial performance [8]. While structural embeddedness broadens the scope of resource acquisition, relational embeddedness deepens resource utilization efficiency. Together, these mechanisms create unique competitive advantages for firms.

Furthermore, scholars have validated the positive effects of network embeddedness on firm innovation and performance from various perspectives [37]. For instance, Feng et al. highlight that green innovation network embeddedness helps firms acquire resources, thereby improving green innovation quality [38–39]. Wei and Zuo argue that makerspace network embeddedness promotes knowledge integration among incubated firms, leading to enhanced innovation performance [40]. Zhang and Xu find that network embeddedness significantly boosts inventors’ innovation performance [41]. Collectively, these studies demonstrate that network embeddedness supports firm innovation and performance through mechanisms such as resource acquisition, knowledge integration, and information flow [42]. In summary, network embeddedness not only broadens firms’ resource acquisition channels but also enhances resource utilization efficiency through strengthened trust and collaboration, laying a solid foundation for sustainable competitive advantages. Therefore, the following hypotheses are proposed:

**H1.** Makerspace network embeddedness has a positive effect on user entrepreneurial performance.
**H1a.** Structural embeddedness has a positive effect on user entrepreneurial performance.
**H1b.** Relational embeddedness has a positive effect on user entrepreneurial performance.

### 2.2. Makerspace network embeddedness and business model innovation

The business model represents the core logic through which firms create value for customers by integrating and utilizing resources, thereby generating revenue [43]. As research has deepened, scholarly attention to business model innovation has gradually shifted from static theoretical analysis to dynamic studies of external environmental characteristics, with network features increasingly becoming a central element of business model innovation [44–45]. For user entrepreneurial ventures embedded in makerspaces, both structural and relational embeddedness not only enable firms to acquire resources efficiently but also provide critical support for business model innovation [46]. Therefore, this study examines the relationship between network embeddedness in makerspaces and the innovative behaviors of user entrepreneurial ventures through the lenses of structural and relational embeddedness.

From the perspective of structural embeddedness, this dimension focuses on a firm’s position within the network and the overall structural characteristics [4]. Higher structural embeddedness is associated with greater network centrality and density, reflecting stronger inter-firm connections within the makerspace [47]. Research indicates that dense network structures facilitate the establishment of collaborative innovation models among user entrepreneurial ventures, providing the heterogeneous information, resources, and capabilities necessary for business model innovation [48]. While sparse networks may offer heterogeneous information, the weaker connections and lack of trust among firms hinder the sustained sharing of resources and information, posing greater challenges to the innovation process [49]. In contrast, dense networks enable firms to acquire resources more efficiently and uncover innovative opportunities [49]. By embedding in makerspaces, user entrepreneurial ventures can establish connections with a broader range of actors, expand resource channels, and gain advantages in resource integration, knowledge sharing, and information transfer, thereby promoting business model innovation [50].

From the perspective of relational embeddedness, this dimension measures the trust, interaction frequency, and durability of connections among members [51]. First, strong relationships imply high levels of trust, which facilitate relational commitment and sustain long-term collaboration. Business models serve as carriers of shared values among collaborating firms, and higher trust levels strengthen cooperative intentions, making it easier to form stable relationships [52]. Second, high trust reduces protective behaviors among members, enhancing the flow of information and resources and enabling firms to integrate and utilize resources more efficiently [4,8]. Finally, strong connections reduce friction between user entrepreneurial ventures and other entities (e.g., governments, communities, and organizations), mitigating risks, avoiding resource waste, and further supporting business model innovation. In summary, structural and relational embeddedness in makerspaces provide critical support for business model innovation in user entrepreneurial ventures by broadening resource acquisition channels and enhancing trust and collaboration. Network embeddedness not only optimizes the efficiency of resource integration and information transfer but also lays a solid foundation for building sustainable competitive advantages, thereby significantly driving business model innovation. Thus, the following hypotheses are proposed:

**H2.** Makerspace network embeddedness positively influences business model innovation in user entrepreneurial ventures.
**H2a.** Structural embeddedness positively influences business model innovation in user entrepreneurial ventures.
**H2b.** Relational embeddedness positively influences business model innovation in user entrepreneurial ventures.

### 2.3. Business model innovation and user entrepreneurial performance

Business model innovation refers to the novel ways in which firms create, deliver, and capture value, achieving internal and external transformations by disrupting traditional operational models, thereby creating greater value and gaining competitive advantages [53]. It is fundamentally a process of “disruptive innovation.” Zott and Amit categorize business model innovation into efficiency-centered and novelty-centered types, both of which provide competitive advantages based on the “cost-value” structure [54]. Research has shown that e-commerce firms, through cross-boundary integration, create new transaction structures that not only generate value for customers but also significantly enhance entrepreneurial performance [55]. Additionally, Wang highlights that entrepreneurial performance is jointly influenced by firm strategy and business models, with business model innovation being a critical source of competitive advantage and performance [56]. In summary, while business model innovation has been shown to influence firm performance, its specific impact on user entrepreneurial performance requires further exploration.

From the perspective of the relationship between business model innovation and user entrepreneurial performance, user entrepreneurial ventures that implement business model innovation place greater emphasis on resource acquisition and information exchange [57]. They excel at drawing growth momentum from external environments and supporting business expansion by enhancing their position within makerspaces, thereby driving performance growth [7,9]. First, innovation in user entrepreneurial ventures often begins with the improvement and optimization of products or services, subsequently triggering transformations in business models [2]. Unlike other forms of entrepreneurship, user entrepreneurship typically originates from the need to address personal demands, which are later transformed into entrepreneurial activities upon recognizing potential commercial value [5]. As a result, user entrepreneurial ventures are more inclined to innovate by designing business models centered on “novelty” or “efficiency,” both of which effectively enhance entrepreneurial performance [2]. Furthermore, business model innovation serves as a key mechanism for commercializing innovative outcomes. Through business model innovation, user entrepreneurial ventures can flexibly adjust their commercialization strategies in response to market dynamics, differentiate themselves from competitors, and establish industry barriers, thereby isolating weaker competitors and securing favorable market positions [3]. This differentiation strategy not only strengthens market competitiveness but also provides robust support for sustained growth in entrepreneurial performance. Therefore, the following hypothesis is proposed:

**H3.** Business model innovation has a positive impact on user entrepreneurial performance.

### 2.4. The mediating role of business model innovation

Business model innovation enables firms to break through existing competitive rules by formulating new development strategies, creating value for both the firm and its stakeholders [57]. For user entrepreneurial ventures, the growth process is inherently a value creation process driven by business model innovation [58]. Grounded in resource-based theory, the survival and development of firms depend on the information and resources they acquire, and business model innovation serves as a critical mediating mechanism connecting resource acquisition and value creation [59]. Specifically, the growth process of user entrepreneurial ventures can be characterized as follows: user entrepreneurs identify business opportunities, exchange information and resources, leverage these resources for innovation, and adjust strategies in response to market changes, ultimately constructing business models with differentiated efficiency. Under resource-constrained conditions, makerspace network embeddedness provides essential support for business model innovation.

From the perspective of structural embeddedness, a higher degree of network embeddedness implies a larger number of members and stronger connections, which not only broadens the channels for resource acquisition but also provides the necessary resource foundation for business model innovation [60]. Enhanced structural embeddedness enables firms to adapt more effectively to environmental changes, acquire entrepreneurial resources needed for innovation, and subsequently expand their core business scale through business model innovation, thereby improving user entrepreneurial performance [61]. From the perspective of relational embeddedness, increased relational embeddedness significantly reduces conflicts among members and strengthens mutual trust, which not only improves the quality of relationships among partners but also expands the scale of collaborative networks [5]. This strong relational network provides robust support for user entrepreneurial ventures to seize innovation opportunities and achieve value creation, further facilitating business model innovation and enhancing entrepreneurial performance. Therefore, the following hypotheses are proposed:

**H4.** Business model innovation mediates the relationship between makerspace network embeddedness and user entrepreneurial performance.
**H4a.** Business model innovation mediates the relationship between structural embeddedness and user entrepreneurial performance.
**H4b.** Business model innovation mediates the relationship between relational embeddedness and user entrepreneurial performance.

### 2.5. The moderating effect of environmental dynamics

Environmental dynamics, as a core characteristic of the external environment, significantly influence the business model innovation process of user entrepreneurial ventures [62]. Environmental dynamics primarily consist of market dynamics and technological dynamics, reflecting the combined effects of changes in consumer demand, the pace of technological iteration, and competitive intensity [63]. This dynamic environment impacts user entrepreneurial ventures in makerspaces through multiple mechanisms, the underlying logic of which warrants in-depth exploration.

First, environmental dynamics constrain business model innovation through the effectiveness of strategic decision-making. In highly uncertain environments, user entrepreneurial ventures often struggle to achieve desired outcomes due to a lack of expertise and experience [64]. This “strategy-environment” misalignment arises from the increased complexity of prediction caused by environmental dynamics, forcing firms to establish dynamic adjustment mechanisms [65]. For instance, during the COVID-19 pandemic, drastic environmental changes pushed many entrepreneurial ventures into survival crises, with only those capable of rapidly adjusting their business models sustaining growth.

Second, environmental dynamics influence innovation performance by reshaping resource acquisition patterns. In turbulent environments, the relational networks between user entrepreneurial ventures and other entities in makerspaces (e.g., communities, governments, and organizations) face reconstruction pressures, manifested as reduced information transmission efficiency and unstable resource acquisition channels [66]. Specifically, highly uncertain competitive environments disrupt existing network relationships, leading to information distortion and hindered resource flows, thereby constraining innovation performance. Additionally, the reliability of market research data decreases in volatile environments, increasing the difficulty of identifying market trends and raising the risk associated with business model innovation.

Third, environmental dynamics moderate organizational cognition, influencing innovation willingness [19]. In low-dynamic environments, the stable returns of existing business models can induce “innovation inertia” within organizations, characterized by reduced environmental sensitivity and insufficient acceptance of and participation in new business models [65]. In such contexts, the role of makerspace network embeddedness in promoting business model innovation is limited [51]. Conversely, in high-dynamic environments, heightened environmental uncertainty increases organizational crisis awareness, fostering a collective recognition of the necessity for innovation. This shift in collective cognition, combined with new information and resources provided by makerspace networks, drives firms to actively pursue business model innovation to adapt to environmental changes.

Finally, environmental dynamics reshape innovation return expectations, influencing innovation investment. In high-dynamic environments, the lifecycle of existing business models shortens, and the potential returns from innovation increase, thereby enhancing firms’ willingness to innovate. Simultaneously, environmental dynamics alter the risk-return structure of innovation: although the risk of innovation failure increases, the opportunity cost of not innovating becomes even higher [65]. This shift in return expectations encourages firms to more actively utilize makerspace network resources and increase investment in business model innovation. Therefore, the following hypotheses are proposed:

**H5.** Environmental dynamics positively moderate the relationship between makerspace network embeddedness and business model innovation.
**H5a.** Environmental dynamics positively moderate the relationship between structural embeddedness and business model innovation.
**H5b.** Environmental dynamics positively moderate the relationship between relational embeddedness and business model innovation.

**Fig 1:**
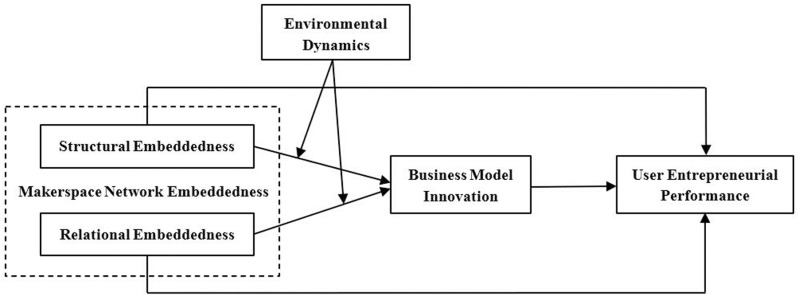
Research model.

Based on the above analysis, this study constructs the research model shown in Fig 1.

## 3. Materials and methods

### 3.1. Sample and data collection

This study investigates the mechanisms through which makerspace network embeddedness affects the entrepreneurial performance of user startups. Following the publication of the “Guiding Opinions of the State Council on Developing Mass Innovation and Entrepreneurship Through Maker Spaces” in 2015, makerspaces have rapidly developed in China. In this context, the study examines how these embedded networks influence user entrepreneurship against the backdrop of national innovation policies. According to the Ministry of Science and Technology’s Torch Program, as of September 2023, Guangdong Province had 1,076 makerspaces, maintaining the top position in the country for six consecutive years. Ministry engineers have noted that the development of makerspaces is closely tied to urban innovation and digitalization levels, with most spaces located in first- and new-first-tier cities in China. Consequently, this study focuses on representative cities within active innovation ecosystems, including first-tier cities such as Beijing, Shanghai, Shenzhen, and Guangzhou, as well as emerging first-tier cities like Hangzhou, Chengdu, and Zhengzhou. These cities were selected due to their high levels of digitalization and innovation, making them ideal locations for exploring the dynamics of makerspace network embeddedness and its impact on entrepreneurial outcomes. Before distributing the survey, the study conducted preliminary exploration to identify potential participants. Through a series of interviews, it was found that founders, mid-level, and senior managers of user startups located in makerspaces and high-tech entrepreneurial parks offered valuable insights into the degree of their business’s network embeddedness and business model innovation capabilities. Therefore, these individuals were selected as the primary participants for this study.

To ensure the representativeness of the sample, a stratified sampling method was employed. The population was first divided into distinct strata based on key characteristics, such as the location of companies and their involvement in digital innovation ecosystems. The target strata included companies operating within maker space platforms, high-tech entrepreneurial parks, and digital incubation bases, specifically those located in regions with relatively advanced digital economies. Cities such as Beijing, Shanghai, Hangzhou, Shenzhen, Guangzhou, and Nanjing were selected due to their strong innovation ecosystems and digital infrastructure, making them ideal for exploring the impact of maker space network embeddedness. Within each stratum, a proportionate random sampling technique was applied to select user entrepreneurs from a range of industries, including internet technology, healthcare, culture and arts, and artificial intelligence. This approach ensured that the sample captured a diverse range of entrepreneurial experiences across different regions and sectors, providing a comprehensive view of user entrepreneurship.

Data collection utilized multiple channels, including online surveys on platforms such as Questionnaire Star, Credamo, WeChat, and offline distribution to founders and mid- to senior-level managers of user startups. Additionally, individuals involved directly or indirectly in entrepreneurial activities, such as students, employees, community workers, and freelancers, were also included based on their engagement with entrepreneurship. The diversity of participants ensured the representativeness and feasibility of the sample. Data collection took place from January 1 to April 30, 2024, yielding a total of 285 survey responses. To ensure data validity, rigorous quality control procedures were employed, including the removal of duplicate and rapidly completed responses that might skew results. A question asking, “Did you start your business due to dissatisfaction with the product or service?” was used to identify user entrepreneurs. After eliminating incomplete or inconsistent responses, a final sample of 245 valid user startup responses remained, yielding an effective response rate of 85.96%. The distribution of the sample is shown in Table 1.

**Table 1:** Sample distribution (n = 245).

Category	Classification	Frequency	Percentage (%)	Category	Classification	Frequency	Percentage (%)
Gender	Male	141	57.6	Founding years	0-1 year	14	5.7
	Women	104	42.4		1-2 years	45	18.4
Age	25 and below	17	6.9		2-3 years	60	24.5
	26-35	145	59.3		3-4 years	61	24.9
	36-45	77	31.4		4 years and above	65	26.5
	Above 45	6	2.4	Industry type	Cultural and artistic	18	7.3
The degree of education	High school and below	86	35.1		Food and beverage	17	6.9
	College degree	91	37.1		Internet technology	35	14.3
	Bachelor’s degree	66	27.0		Logistics and distribution	52	21.2
	Graduate education	2	0.8		Sports and health	46	18.8
Company size	0-10	104	42.4		Agricultural products	36	14.7
	11-20	61	25.0		Outdoor sports	13	5.3
	21-30	76	31.0		Education and training	16	6.6
	Above 30	4	1.6		Other	12	4.9

This study was conducted in accordance with the Declaration of Helsinki and approved by the Institutional Review Board of the School of Economics and Management at Guangxi Normal University. Additionally, participants were required to provide written informed consent, and all surveys were completed anonymously to ensure participants’ privacy. These procedures were strictly followed throughout the data collection process to ensure compliance with ethical standards.

### 3.2. Measures

To ensure the reliability and validity of the scale, this study employs authoritative scales developed by both domestic and international scholars and adapts them to the specific context of entrepreneurship in China. In measuring makerspace network embeddedness, based on the studies of Granovetter [6] and Stevens and van Schaik [31], we divide network embeddedness into two dimensions—structural embeddedness and relational embeddedness—according to network embeddedness theory. In this context, we have modified the original scales to better align with the particular environment of Chinese makerspace. For example, when measuring structural embeddedness, we included items such as “this company holds a central position in the makerspace network” and “close relationships among members in the makerspace network where this company is located,” to more accurately reflect the network position and relational ties of user entrepreneurial firms within the makerspace. In measuring relational embeddedness, we added items such as “there is mutual trust between this company and other members and organizations within the makerspace” and “this company has established long term collaborative relationships with other members or organizations within the makerspace,” to better capture the interaction and trust levels between the firm and other members or organizations.

In measuring business model innovation, we drew on the research of Zott and Amit [44] and Wei et al. [58] and selected six items after refinement, such as “the business model reduces various costs for business partners” and “the business model offers a new combination of products, services, and information,” to comprehensively assess the business model innovation capabilities of user entrepreneurial firms in makerspaces. For measuring user entrepreneurial performance, we referenced the work of Covin et al. [66], Wiklund [67], and Chrisman et al. [68], selecting seven items such as “this company’s entrepreneurial products and services have high profitability and profit margins,” “the number of employees in this company is continuously growing,” and “compared to other competitors, this company’s entrepreneurial products and services are updated quickly and have a wide range of product offerings,” to comprehensively evaluate the survival, growth, and innovation performance of user entrepreneurial firms.

In measuring environmental dynamics, drawing on the studies of Miller [69] and Sheng et al. [70], we address both market dynamics and technological dynamics. We adjusted the original scales accordingly. For example, in measuring market dynamics, we selected items such as “consumer product demands and preferences have been constantly changing within this company’s industry,” to better reflect the dynamic changes in market demand. In measuring technological dynamics, we selected items such as “most technological developments in the industry involve a fundamental change from existing technologies,” to better capture the dynamic nature of technological advancements.

To control for common method bias, we implemented both pre- and post-control measures. The pre-control measures involved ensuring respondent anonymity and providing clear instructions to minimize social desirability bias, which helped reduce potential biases in data collection. The post-control measures included performing a Harman’s single-factor test to assess the extent of common method bias in the data. This test helps confirm that the results are not unduly influenced by common method bias, ensuring the robustness and validity of our findings. Each variable was measured on a 5-point Likert scale ranging from 1 “strongly disagree” to 5 “strongly agree”. Specific items are presented in Table 2.

**Table 2:** Results of reliability and validity test (n = 245).

Variable	Item	Factor Loading	KMO	Cronbach’ s α	CR	AVE
MSE	MSE1. A large number of members in the makerspace network where this company is located.	0.752	0.748	0.892	0.797	0.567
	MSE2. This company holds a central position in the makerspace network.	0.728				
	MSE3. Close relationships among members in the makerspace network where this company is located.	0.778				
MRE	MRE1. This company frequently collaborates and communicates with other members and organizations within the makerspace.	0.749	0.722	0.850	0.834	0.627
	MRE2. There is mutual trust between this company and other members and organizations within the makerspace.	0.768				
	MRE3. This company has established long-term collaborative relationships with other members or organizations within the makerspace.	0.855				
BMI	BMI1. The business model reduces various costs for business partners.	0.761	0.920	0.917	0.910	0.630
	BMI2. The business model enables faster and more efficient transactions.	0.720				
	BMI3. The business model offers a new combination of products, services, and information.	0.747				
	BMI4. The business model allows for more business partners to participate, creating better products.	0.907				
	BMI5. This company tends to innovate by following market leaders.	0.820				
	BMI6. The business model attempts to use new methods to connect trading partners.	0.793				
UEP	UEP1. This company’s entrepreneurial products and services have high profitability and profit margins.	0.755	0.946	0.940	0.913	0.599
	UEP2. This company’s entrepreneurial products and services have a high market share.	0.778				
	UEP3. The number of employees in this company is continuously growing.	0.776				
	UEP4. This company has strong profit potential in the future.	0.744				
	UEP5. This company’s market share for future products is expected to expand further.	0.751				
	UEP6. Compared to other competitors, this company’s entrepreneurial products and services are updated quickly and have a wide range of product offerings.	0.832				
	UEP7. Compared to other competitors, this company has more innovative ideas and implements them in production.	0.777				
ED	ED1. Consumer product demands and preferences have been constantly changing within this company’s industry.	0.787	0.832	0.879	0.839	0.565
	ED2. Consumers within this company have been consistently seeking new products and services.	0.717				
	ED3. The industry’s technological changes are happening rapidly.	0.735				
	ED4. Most technological developments in the industry involve a fundamental change from existing technologies.	0.767				

Abbreviations: MSE, makerspace structural embeddedness; MRE, makerspace relational embeddedness; BMI, business model innovation; UEP, user entrepreneurial performance; ED, environmental dynamics. Same below.

### 3.3. Estimation technique

This study employs hierarchical regression analysis to examine the relationships involving all hypotheses related to direct effects [71], and uses the “PROCESS” macro [72] with the bootstrap method to test for mediation effects. Firstly, Hierarchical regression analysis is employed to examine the direct relationships between makerspace network embeddedness (including structural and relational embeddedness), business model innovation, and user entrepreneurial performance. This method is chosen because it allows for a stepwise introduction of predictor variables while controlling for confounding factors. In the first step, control variables are added to account for external influences that could affect the outcomes. Then, we progressively introduce the predictor variables—namely structural embeddedness, relational embeddedness, and business model innovation—to examine their individual and cumulative effects on user entrepreneurial performance (hypotheses 1a, 1b, and 3). This incremental approach ensures that we can systematically assess the influence of each variable while controlling for others.

Next, hierarchical regression analysis is used to explore the impact of makerspace network embeddedness on business model innovation (hypotheses 2a and 2b). By adding control variables first, followed by the predictor variables, this method allows for a clear understanding of the direct effects of the embeddedness dimensions on business model innovation. Subsequently, we used multiple regression analysis to examine the mediation and moderation effects (hypotheses 4a, 4b, 5a, and 5b).

Finally, we employed the “PROCESS” macro (Preacher and Hayes, 2008) again to test for mediation effects and ensure robustness, which is specifically designed to test complex indirect relationships, including mediation and moderation. The advantage of using the “PROCESS” macro with the bootstrap method lies in its ability to generate more robust and reliable results, as it estimates the coefficients in the mediation paths while providing bias-corrected confidence intervals through 5,000 resamples. This bootstrapping approach is superior to traditional methods like the Sobel test, as it ensures greater reliability and power in detecting mediation effects. As a result, this technique has been increasingly adopted in recent entrepreneurship and innovation research [71,73]. For the mediation analysis, we input makerspace structural and relational embeddedness as independent variables, control variables as covariates, business model innovation as the mediator, and user entrepreneurial performance as the dependent variable.

## 4. Results

### 4.1. Reliability and validity test

We utilized the statistical software SPSS 27.0 and Amos 25.0 to test the reliability and validity of the variable scales, and the results are shown in Table 2. According to the results of the reliability test, the Cronbach’s α of each variable is greater than 0.7, and the composite reliability (CR) values are greater than 0.7, indicating that the adopted scales have good reliability. Cronbach’s α is used to measure the internal consistency of a scale, and values above 0.7 indicate good reliability, meaning that the items within a scale are consistently measuring the same construct. CR is another indicator of internal consistency, specifically for latent variables, with values greater than 0.7 signifying strong reliability. The results of exploratory factor analysis (EFA) showed that the KMO values of structural embeddedness, relational embeddedness, business model innovation, user entrepreneurial performance, and environmental dynamics ranged from 0.722 to 0.946, which were all greater than 0.7 and P < 0.001, indicating that the data is suitable for factor analysis. Kaiser-Meyer-Olkin (KMO) measures the sampling adequacy for factor analysis, and values above 0.7 indicate that the data is appropriate for factor analysis.

Further, as shown in Table 3, the model was tested by the maximum likelihood estimation method, and the fit indexes of the research model were: CMIN/DF = 1.643 < 3, RMSEA = 0.011 < 0.08, RMR = 0.021 < 0.05, and CFI = 0.960, IFI = 0.962, and TLI = 0.954, all greater than the standard value of 0.9, indicating that the research model fitted well with the observational data as a whole. In addition, convergent validity was tested by confirmatory factor analysis (CFA), and the standardized factor loadings (FL) of each question item were all greater than 0.6. The average variance extracted (AVE) for each construct was greater than 0.5, indicating good convergent validity and structural validity of each variable. FL reflects the strength of the relationship between each item and its latent construct, with values greater than 0.6 indicating that the item strongly represents the latent construct. AVE is used to assess the amount of variance captured by a construct, with values greater than 0.5 indicating good convergent validity, meaning that the construct explains more than half of the variance in its indicators. Moreover, as shown in Table 4, the square roots of the AVE values were all greater than the correlation coefficients of the variables, indicating good discriminant validity among the variables.

**Table 3:** Structural equation model fitting results.

Index	CMIN/DF	RMSEA	RMR	CFI	IFI	TLI
Acceptable criteria	< 3	< 0.08	< 0.05	> 0.9	> 0.9	> 0.9
Data results	1.643	0.011	0.021	0.960	0.962	0.954

Notes: N = 245. CMIN/DF, Chi-Square/Degrees of freedom; RMSEA, Root Mean Square Error of Approximation; RMR, Root Mean Square Residual; CFI, Comparative Fit Index; IFI, Incremental Fit Index; TLI, Tucker-Lewis Index.

**Table 4:** Descriptive statistics and correlation analysis results (n = 245).

		Mean	SD	1	2	3	4	5	6	7	8	9
1	Gender	1.551	0.498	–								
2	Age	2.270	0.634	0.114	–							
3	Education level	1.930	0.811	-0.041	0.057	–						
4	Founding years	3.491	1.218	-0.217^**^	0.027	-0.092	–					
5	MSE	3.918	0.924	0.058	0.074	0.013	-0.029	**0.753**				
6	MRE	3.437	0.937	0.090	0.080	0.057	-0.108	0.437^**^	**0.792**			
7	BMI	3.390	0.890	-0.051	0.120	0.084	0.054	0.414^**^	0.229^**^	**0.794**		
8	UEP	3.557	0.929	0.008	0.132^*^	0.127^*^	0.013	0.441^**^	0.402^**^	0.411^**^	**0.774**	
9	ED	3.174	0.932	0.078	0.072	0.018	-0.049	0.341^**^	0.337^**^	0.242^**^	0.310^**^	**0.752**

Notes: SD, standard deviation; *P < 0.05, **P < 0.01, two-tailed test. The diagonal is the AVE square root of the core variable.

### 4.2. Common method bias (CMB) test

This study employed pre-control and post-control methods to reduce the risk of common method bias. In terms of pre-control, the latent variables were measured by conducting an anonymous survey, designing multi-question items of different types and sources, and reducing artificial covariance by changing the order of the questions. As for post-control, Harman’s single-factor test was used to examine common method bias. The results revealed the presence of five factors with eigenvalues greater than 1. However, the unrotated first factor explained only 27.094% of the variance, which did not exceed the commonly used threshold of 40%, which indicated that there was no significant common method bias problem.

### 4.3. Correlation analysis

Descriptive statistics and correlation analysis are shown in Table 4. The correlation coefficients between all variables were less than 0.7, indicating the absence of significant multicollinearity. The covariance diagnostic results show that the Variance Inflation Factor (VIF) of each variable is between 1.018 and 1.424, which is less than 5, further ruling out the existence of significant multicollinearity. In addition, there is a significant positive correlation between the structural embeddedness, relational embeddedness, and business model innovation of the makerspace (r = 0.414, p < 0.01; r = 0.229, p < 0.01); and there is a significant positive correlation between business model innovation and user entrepreneurial performance (r = 0.411, p < 0.01), providing preliminary support for further regression analysis.

### 4.4. Hypothesis tests

#### 4.4.1. Main effects test.

We conducted a multiple regression analysis using SPSS 27.0 to test the hypotheses related to the direct effects. The results of the regression analysis are shown in Table 5. Model 5 shows that structural embeddedness in makerspaces has a significant positive effect on user entrepreneurial performance (β=0.434, P < 0.001); Model 6 shows that relational embeddedness has a significant positive effect on user entrepreneurial performance (β=0.397, P < 0.001). Therefore, hypotheses H1a and H1b are supported, confirming H1. This indicates that user entrepreneurial ventures can continuously acquire valuable information and resources through structural and relational embeddedness in makerspaces, and utilize these resources to expand production scale, market share, and accelerate the iteration speed of entrepreneurial products, thereby driving continuous growth and innovation of enterprises. Model 2 demonstrates that structural embeddedness in makerspaces has a significant positive effect on business model innovation (β=0.412, P < 0.001); Model 3 shows that relational embeddedness has a significant positive effect on business model innovation (β=0.230, P < 0.001). Consequently, hypotheses H2a and H2b are confirmed, establishing H2. This implies that user entrepreneurial ventures can promote resource integration, knowledge sharing, and information transmission through structural and relational embeddedness in makerspaces, effectively combining existing products, services, and information resources to achieve business model innovation. As indicated by Model 7, business model innovation has a significant positive effect on user entrepreneurial performance (β=0.395, P < 0.001), thus supporting hypothesis H3. This suggests that business model innovation can enhance the value acquisition and creation capabilities of user entrepreneurial ventures, thereby promoting the growth of their entrepreneurial performance.

**Table 5:** Results of the regression analysis.

Variable	BMI			UEP						
	Model 1	Model 2	Model 3	Model 4	Model 5	Model 6	Model 7	Model 8	Model 9	Model 10
Control variable										
Gender	-0.051	-0.070	-0.066	0.003	-0.017	-0.022	0.023	0.001	-0.001	-0.011
Age	0.120	0.092	0.104	0.124	0.095	0.096	0.077	0.071	0.062	0.062
Education	0.079	0.075	0.068	0.121	0.118	0.103	0.090	0.098	0.081	0.089
Founding years	0.047	0.056	0.068	0.021	0.030	0.058	0.002	0.016	0.036	0.036
Independent variable										
MSE		0.412^***^			0.434^***^			0.328^***^		0.232^***^
MRE			0.230^***^			0.397^***^			0.323^***^	0.241^***^
Mediating variable										
BMI							0.395^***^	0.257^***^	0.321^***^	0.242^***^
*R^2*	0.026	0.195	0.078	0.032	0.219	0.186	0.184	0.272	0.281	0.318
*Adjusted R^2*	0.010	0.178	0.058	0.016	0.203	0.169	0.167	0.254	0.262	0.298
*F*	1.615	11.565^***^	4.031^**^	1.991	13.423^***^	10.914^***^	10.782^***^	14.856^***^	15.474^***^	15.807^***^

Notes: **P < 0.01, ***P < 0.001, two-tailed test.

Abbreviations: MSE, makerspace structural embeddedness; MRE, makerspace relational embeddedness; BMI, business model innovation; UEP, user entrepreneurial performance. Same below.

The results suggest that both structural and relational embeddedness are critical factors influencing the performance and innovation of user entrepreneurial ventures. These findings underscore the significance of strong network connections within makerspaces, as they facilitate user entrepreneurial ventures’ access to essential resources and support networks. Through structural embeddedness, firms gain access to vital resources such as information, capital, and technology, which are crucial for business model innovation. Relational embeddedness, on the other hand, fosters trust and collaboration among network members, enabling the sharing of knowledge and the formation of valuable partnerships.

This aligns with the resource-based view, which posits that access to valuable and rare resources is a key driver of innovation and competitive advantage. According to resource-based view, the more deeply a firm is embedded in its network, the greater its opportunities to acquire and integrate resources, which enhances its ability to innovate and improve its performance. The results indicate that user entrepreneurial ventures leverage these embedded resources—whether through enhanced knowledge flow, improved resource allocation, or collaborative innovation efforts—to strengthen their market position and foster continuous innovation. In practical terms, this suggests that user entrepreneurial ventures within makerspaces should not only focus on building strong network ties but also strategically leverage these relationships to access valuable resources and knowledge that can drive innovation. By integrating these resources into their business models, ventures can ensure sustained growth and long-term innovation. This emphasizes the vital role of embeddedness in not only improving immediate performance but also in securing the long-term sustainability and competitive advantage of user entrepreneurial ventures.

#### 4.4.2. Mediating effects test.

The results of the mediating test are shown in Table 5. In model 8, both structural embeddedness and business model innovation of the makerspace are included in the regression equation, and the results are positive and significant, but the regression coefficient of structural embeddedness decreases from 0.434 to 0.328, which suggests that the business model innovation (β=0.257, p < 0.001) plays a mediating role between the structural embeddedness and the performance of user entrepreneurship, and the hypothesis H4a is verified. This suggests that user entrepreneurial enterprises with strong structural embeddedness in the makerspace are able to reduce all costs of entrepreneurship and provide new products, services and information combinations by discovering breakthroughs in business model innovation through collaborative innovation with other innovators, thus enhancing entrepreneurial performance. In model 9, both relational embeddedness and business model innovation are included in the regression equation, and the results are positive and significant, but the regression coefficient of relational embeddedness decreases from 0.397 to 0.323, which indicates that business model innovation (β=0.321, p < 0.001) plays a mediating role between relational embeddedness and user entrepreneurial performance, and the hypothesis H4b is verified, and so H4 is supported. This suggests that user entrepreneurial ventures with strong relational embeddedness in the makerspace achieve higher entrepreneurial performance by forming mutual trust with other subjects, constructing associative information systems and resource network links, and promoting business model innovation. In model 10, structural, relational embeddedness and business model innovation are put into the regression equation at the same time, and the results are all positive and significant, but the regression coefficient of structural embeddedness decreases from 0.434 to 0.232, and the regression coefficient of relational embeddedness decreases from 0.397 to 0.241. This shows that business model innovation (β=0.242, p < 0.001) plays a mediating role between makerspace network embeddedness and user entrepreneurial performance, and thus hypothesis H4 is still supported. This suggests that user-entrepreneurial enterprises with strong network embeddedness in the makerspace construct differentiated and efficient business models through resource acquisition and integration and network relationship building to reduce entrepreneurial risk, improve their market competitiveness, and thus achieve improved entrepreneurial performance.

These results highlight the crucial role of business model innovation as a mediator in the relationship between both structural and relational embeddedness and user entrepreneurial performance. The reduction in the direct effects of embeddedness after accounting for the mediating role of business model innovation underscores the importance of innovation in translating the advantages of embeddedness into tangible improvements in user entrepreneurial performance. In practical terms, this suggests that makerspace ventures should not only focus on building strong networks but also actively foster a culture of innovation to fully leverage the benefits of their embeddedness. Furthermore, these findings emphasize the strategic role of collaborative innovation within networks as a pathway to reduce entrepreneurial risks, enhance market competitiveness, and ultimately drive business success. Theoretical models of entrepreneurship and innovation can incorporate these insights to better explain how embeddedness influences firm outcomes through innovation processes.

#### 4.4.3. Moderating effects test.

Hierarchical regression analysis was utilized to test the moderating effect of environmental dynamics, and the results are presented in Table 6. The results of model 12 show that the coefficient of the interaction term between structural embeddedness and environmental dynamics is significantly positive (β=0.285, P < 0.01), indicating that environmental dynamics positively and significantly moderates the relationship between structural embeddedness and business model innovation, and hypothesis H5a is supported. The results of model 14 show that the coefficient of the interaction term between relational embeddedness and environmental dynamics is significantly positive (β=0.214, P < 0.01), indicating that environmental dynamics positively and significantly moderates the relationship between relational embeddedness and business model innovation, and hypothesis H5b is supported, and thus hypothesis H5 is supported. This suggests that user entrepreneurial firms with strong network embeddedness (structural and relational embeddedness) in the makerspace are able to perceive the uncertainty of changes in the technological and market environments, and attempt to adapt to dynamically changing environments by integrating and utilizing entrepreneurial resources and expanding their product and service business models, thereby advancing the innovation of their business models.

**Table 6:** Results of the moderating effect test for environmental dynamics.

Variable	BMI						
	Model 1	Model 2	Model 11	Model 12	Model 3	Model 13	Model 14
Control variable							
Gender	-0.051	-0.070	-0.075	-0.077	-0.066	-0.073	-0.068
Age	0.120	0.092	0.087	0.090	0.104	0.096	0.094
Education	0.079	0.075	0.074	0.074	0.068	0.069	0.067
Founding years	0.047	0.056	0.059	0.065	0.068	0.069	0.076
Independent variable							
MSE		0.412^***^	0.374^***^	0.330^***^			
MRE					0.230^***^	0.169^***^	0.184^***^
Moderating variable							
ED			0.215^*^	0.247^**^		0.186^**^	0.193^**^
Interaction term							
MSE * ED				0.285^**^			
MRE * ED							0.214^**^
*R^2*	0.026	0.195	0.206	0.208	0.078	0.148	0.172
*Adjusted R^2*	0.010	0.178	0.186	0.184	0.058	0.086	0.085
*F*	1.615	11.565^***^	10.320^***^	10.878^***^	4.031^**^	4.811^***^	5.255^***^

Notes: *P < 0.05, **P < 0.01, ***P < 0.001, two-tailed test.

The moderating effects test underscores the dynamic nature of user entrepreneurial ventures in makerspaces. Environmental dynamics, particularly changes in technology and market conditions, significantly influence how embeddedness impacts business model innovation. These results suggest that user entrepreneurial ventures with strong network ties are more adaptable and capable of navigating uncertain environments. In practical terms, this means that ventures must remain attuned to external shifts, leveraging their embeddedness within makerspaces to not only respond to these changes but also proactively innovate their business models. These findings contribute to a deeper understanding of how environmental factors interact with network embeddedness to shape the success of user entrepreneurial ventures. This interplay between internal network resources and external environmental factors provides valuable insights for both theory and practice in the field of entrepreneurship.

#### 4.4.4. Robustness test.

To enhance the reliability of the conclusions, this study further conducted a robustness test of the mediation effect using the bootstrap method, with 5000 resamples. The bias-corrected percentile method was employed to calculate the 95% confidence intervals, and the results are presented in Table 7. The bias-corrected bootstrap confidence interval for the indirect effect of Path 1 at the 95% confidence level was (0.106, 0.573), with the interval not containing zero and a p-value less than 0.001, indicating that Hypothesis H4a is supported. Similarly, the bias-corrected bootstrap confidence interval for the indirect effect of Path 2 at the 95% confidence level was (0.123, 0.649), with the interval not containing zero and a p-value less than 0.001, indicating that Hypothesis H4b is supported. These findings confirm the mediation effect and are consistent with the previous conclusions.

**Table 7:** Results of bootstrap mediating path test.

Hypothesis	Mediating path	Path coefficient	S.E.	95% Confidence interval	P	Conclusion
H4a	Path 1 = MSE → BMI → UEP	0.337	0.119	[0.106, 0.573]	***	Supported
H4b	Path 2 = MRE → BMI → UEP	0.386	0.134	[0.123, 0.649]	***	Supported

Notes: *** P < 0.001.

The robustness test results provide additional confirmation of the stability and reliability of the mediation effects observed in the previous tests. By utilizing a bootstrap method with resampling, the findings become even more robust and less susceptible to random errors, further validating the mediation effects of business model innovation. This strengthens the credibility of the conclusions and ensures that the relationships identified in the models are not only statistically significant but also practically meaningful. These additional tests provide a higher level of confidence in the study’s findings and reinforce the proposed theoretical framework, offering valuable insights for future research in the field of entrepreneurship and innovation.

## 5. Discussion

Based on network embeddedness theory and resource-based theory, this study employs the hierarchical regression and bootstrap methods to examine the relationship between different dimensions of makerspace network embeddedness and the entrepreneurial performance of user entrepreneurial firms, as well as the mediating role of business model innovation and the moderating effect of environmental dynamics. The empirical findings are as follows.

(1) Makerspace network embeddedness significantly enhances user entrepreneurial performance. This study confirms that both structural and relational embeddedness in makerspaces significantly contribute to enhancing user entrepreneurial performance, aligning with existing research. Zhou and Li, from the perspective of entrepreneurial learning capabilities, suggest that user entrepreneurial ventures embedded in makerspace networks can improve their entrepreneurial learning capabilities, thereby enhancing entrepreneurial performance. They argue that makerspace network embeddedness moderates the relationship between entrepreneurial performance and learning capabilities [8]. This study extends this perspective by positing that makerspace network embeddedness enhances users’ ability to identify opportunities and acquire resources. From a resource-based view, structural embeddedness facilitates the integration of both internal and external resources, such as funding, technology, and information, which in turn provides more comprehensive and high-quality services to entrepreneurs. This improved resource integration not only boosts entrepreneurial performance but also contributes to the overall development of the entrepreneurial ecosystem [43,74]. The larger the makerspace, the more user entrepreneurial ventures can differentiate their relationships with other members and institutions, gaining access to critical resources that drive innovative opportunities and technologies, thereby improving entrepreneurial performance [75]. In contrast, relational embeddedness facilitates more efficient information sharing and knowledge exchange among members. This rapid flow of information accelerates resource sharing, idea generation, and collaboration, fostering an environment of communication and innovation within the makerspace. As a result, it accelerates the idea and project incubation processes, facilitating the formation of user entrepreneurial communities [76]. These strong, collaborative relationships help entrepreneurs acquire the resources needed for innovation, test their products, gather feedback, and continuously improve their offerings, thereby increasing the speed of product updates and enhancing overall entrepreneurial performance.
(2) Makerspace network embeddedness has a significant positive impact on business model innovation. Various factors influence business model innovation, including internal and external environments, entrepreneurial capabilities, and resource integration capabilities [77,78]. From the perspective of network embeddedness, Guo et al. found that network embeddedness has a facilitating effect on the growth of science and technology-based enterprises, and science and technology-based enterprises acquire information and resources through network embeddedness to promote business model innovation [79]. This study aligns with previous research, confirming that both structural and relational embeddedness in makerspaces significantly promote business model innovation. Makerspaces with higher structural embeddedness typically possess stronger innovation capabilities, continuously fostering the generation and implementation of new technologies and products, and accelerating the transformation of entrepreneurial outcomes [75,76]. User-entrepreneurs residing in a makerspace are able to establish symbiotic relationships with different entrepreneurial entities and realize the sharing of resources and information [8,15]. In addition, relational embeddedness strengthens the trust between symbiotic subjects, and the close interaction and cooperation between members can stimulate innovation inspiration and entrepreneurship, promote mutual collaborative innovation, and help to realize the maximization of the utility of the network resources, and improve the user entrepreneurial enterprise’s perception and adaptive ability to environmental changes [75,80]. Moreover, the network embeddedness of the makerspace enriches the resources available to user entrepreneurs and effectively integrates the information system in the makerspace with the support of government policies, which effectively promotes business model innovation.
(3) Business model innovation has a significant positive effect on user entrepreneurial performance. In the era of digital economy, the emergence and popularization of the Internet provide a wide space for business model innovation. A group of new Internet-based entrepreneurial enterprises such as Amazon and eBay have achieved tremendous development in a short period of time through business model innovation [81]. Especially in the post-epidemic period, the success of these new startups has had a profound impact on traditional enterprises, inspiring them to rethink the way of profitability and further scrutinize the importance of business model innovation in a period of turbulence and change [79,80]. At the same time, some scholars have pointed out that business model innovation can also have a positive impact on user entrepreneurial performance, including survival performance, growth performance, and innovation performance [81,82]. First, innovative business models can improve the survival rate of user entrepreneurship, reduce the risk of failure, and increase the sustaining ability of the enterprise [82]. Second, new business models bring higher competitive advantage and market share, which motivate user entrepreneurs to expand, improve profitability and achieve sustainable development [81,82]. Finally, innovative business models can motivate user entrepreneurs to make more technological, product or service innovations to meet market demands and provide innovative solutions, thus realizing improved innovation performance.
(4) Business model innovation plays a partial mediating role between makerspace network embeddedness and user entrepreneurial performance, contributing to bridging gaps in the literature related to the mechanisms that explain how network structures within makerspaces support entrepreneurial success. According to network embeddedness theory, the interconnections within a network provide firms with access to diverse resources and collaborative opportunities that they may not have within their direct control. Makerspaces, through their network embeddedness, provide suitable partners and resource access channels, assisting user entrepreneurial firms in achieving business model innovation and subsequently enhancing entrepreneurial performance [75,76]. From a resource-based view, makerspaces offer a rich environment that strengthens firms’ resource base by expanding avenues for resource acquisition, enhancing their adaptability, and improving their capacity for innovation. These factors are essential for the integration of resources required to develop and refine business model innovations [83]. Additionally, high-intensity relational embeddedness, a key dimension of network embeddedness, reduces internal conflicts, fosters trust, and strengthens partnerships. Such relational dynamics are fundamental in promoting mutual growth and collaboration, which in turn create favorable conditions for firms to seize innovation opportunities and generate value [75,79]. The relational aspect of embeddedness thus supports the resource integration and dynamic capabilities necessary for continuous business model evolution. In sum, the network embeddedness of makerspaces not only facilitates business model innovation but also strengthens the competitive advantage of entrepreneurial firms, allowing them to achieve sustained performance returns. This alignment with both network embeddedness theory and the resource-based view underscores the critical role of makerspace networks in driving innovation and entrepreneurial success.
(5) Environmental dynamics can positively modulate the relationship between network embeddedness and business model innovation in makerspaces. Scholars have found that the speed of change in the external environment affects the ability of enterprises to absorb knowledge. When the technological environment becomes more uncertain and the speed of change increases, it becomes more challenging for enterprises to recoup lost knowledge and utilize existing knowledge to drive business model innovation [11,79,83]. This study, based on network embeddedness theory, confirms that environmental dynamics positively modulate the effects of structural and relational embeddedness in makerspaces on business model innovation [84]. Higher environmental dynamics signify that the external environment is subject to rapid and substantial changes, increasing uncertainty [84]. Different studies have found contrasting results regarding the role of environmental dynamics in business model innovation. The reason for these discrepancies may stem from methodological differences. For instance, some studies used cross-sectional data, while others used longitudinal data, leading to different insights about the effect of environmental changes over time. Moreover, contextual factors such as industry, geographical location, and market maturity may explain these varied results. For example, studies conducted in highly competitive, technology-driven markets may find a stronger impact of environmental dynamics compared to those in more stable or regulated environments. In the context of user entrepreneurs in makerspaces, these high environmental dynamics are particularly challenging. User entrepreneurs often lack relevant management experience and knowledge reserves. The more dynamic the environment, the more difficult it becomes for them to predict future trends and develop effective strategic solutions [85]. As a result, user entrepreneurs are increasingly reliant on the resources and partnerships provided by makerspaces to access critical information, technical support, and networks to respond to the rapidly changing market demands [86]. For user-entrepreneurs, recognizing and responding to environmental dynamics is crucial. It allows them to better adapt to changes in both internal and external environments [84,86]. However, the structural and relational embeddedness within makerspaces provides these entrepreneurs with a wealth of resources and partners, which help them better adapt, innovate their business models, and enhance both personal and organizational adaptability and innovativeness. This gives them a competitive edge in an increasingly volatile market.

## 6. Summary

### 6.1. Conclusion

This study explores the impact of makerspace network embeddedness on user entrepreneurial performance, highlighting the mediating role of business model innovation and the moderating effect of environmental dynamics. Drawing on network embeddedness theory and the resource-based view, our findings demonstrate that both structural and relational embeddedness significantly enhance business model innovation, which in turn fosters user entrepreneurial performance. The results reveal that business model innovation partially mediates the relationship between makerspace network embeddedness and entrepreneurial performance, underscoring the importance of leveraging network resources for sustainable growth. Furthermore, environmental dynamics positively moderate the effect of network embeddedness on business model innovation, suggesting that firms embedded in makerspaces must remain agile in responding to external uncertainties.

These findings contribute to the literature by elucidating the mechanisms through which makerspace networks influence entrepreneurial outcomes, expanding the theoretical understanding of network embeddedness and business model innovation in dynamic environments. From a practical perspective, user entrepreneurs should actively cultivate both structural and relational embeddedness within makerspaces to maximize resource acquisition, collaboration opportunities, and innovation potential. Additionally, policymakers and makerspace managers should foster an ecosystem that supports network integration and business model innovation to enhance the long-term success of user entrepreneurial ventures.

### 6.2. Theoretical contributions

First, this study innovatively constructs a model of the relationships among makerspace network embeddedness, business model innovation, and user entrepreneurial performance from the perspectives of network embeddedness and business model innovation, revealing the mechanisms by which makerspace network embeddedness influences user entrepreneurial performance. Although existing studies have explored the transformation mechanism from user innovation to user entrepreneurship from the perspectives of user community, user identity, and entrepreneurial capability, there is a lack of research on user entrepreneurship from the perspectives of network embeddedness and business model innovation. Focusing on user entrepreneurship enterprises, this study examines the relationship between network embeddedness, business model innovation and user entrepreneurship performance in makerspaces, expanding the scope of research on entrepreneurial performance objects and enriching theoretical research on the field of user entrepreneurship. In addition, this study extends the application of resource-based theory. Resource-based theory focuses on how firms utilize their unique resources to build competitive advantage, but often neglects the location of these resources in the network and their dynamic acquisition process. By introducing the network embeddedness perspective, this study reveals how firms in a makerspace acquire resources and information by embedding themselves in the network and how they utilize these resources for business model innovation to enhance entrepreneurial performance, which not only complements the resource-based theory’s understanding of resource acquisition and utilization, but also provides a new perspective for studying the dynamics of resources in a crowdsourced makerspace. By combining network embeddedness and resource-based theory, this study demonstrates how user-entrepreneurial firms can construct and utilize resources in complex network environments to achieve business model innovation and thus enhance entrepreneurial performance. This integration not only deepens the understanding of resource-based theory, but also provides a theoretical basis for how user entrepreneurial firms in makerspaces can better leverage their network resources.

Second, this study reveals the mechanisms by which different dimensions of network embeddedness in makerspaces influence business model innovation. Previous studies have focused on the impact of structural embeddedness on entrepreneurs’ resource acquisition and information transfer, emphasizing the impact of structural indicators such as the degree of connectivity and agglomeration between nodes in the network on entrepreneurs’ innovation activities. However, these studies often overlook the potential impact of the quality and depth of relational embeddedness on business model innovation. Based on resource-based theory, this study finds that network embeddedness in makerspaces helps firms to acquire the resources and information necessary for survival and development, both of which drive business model innovation and thus enhance user entrepreneurial firm performance. This study not only confirms the mechanisms of different dimensions of makerspace network embeddedness, especially the focus on relational embeddedness, but also reveals the significant impact of relational embeddedness on business model innovation. Relational embeddedness emphasizes factors such as trust, reciprocity, and frequency of interaction between nodes in a network, which significantly influence the quality and availability of resources and information. By examining relational embeddedness in depth, this study enriches the understanding of the mechanisms influencing user entrepreneurs’ business model innovation in a makerspace, complements existing research, and provides theoretical support for how user entrepreneurial firms can more effectively utilize network resources in a makerspace. Therefore, this study not only expands the scope of application of resource-based theory, but also provides new perspectives for understanding the complex impact of a makerspace on business model innovation.

Third, this study uncovers the moderating effects of environmental dynamics on the relationship between makerspace structural and relational embeddedness and business model innovation. Unlike previous research that primarily focuses on the impact of internal structure and relationships within makerspaces on business model innovation, this study further considers how external environmental dynamism adjusts this relationship. This comprehensive perspective brings new theoretical insights to related fields and expands the boundaries of research on the relationship between the structure and relational embeddedness of makerspaces and business model innovation. Moreover, the findings resonate with network embeddedness theory, highlighting that environmental dynamics, as an external factor, influences the formation and evolution of internal structures and relationships within makerspaces, subsequently affecting the pathways and outcomes of business model innovation. In the digital economy era, the emergence and widespread use of the Internet provide vast opportunities for business model innovation. Post-pandemic, enterprises face increased uncertainty and challenges, necessitating greater flexibility and adaptability to cope with rapidly changing external environments. This study also aids in understanding how, in a post-pandemic era characterized by swift external changes, user entrepreneurial firms must develop flexibility and adaptability to strengthen structural and relational embeddedness within makerspaces, thereby achieving business model innovation. Therefore, considering environmental dynamics not only deepens but also extends the network embeddedness theory.

### 6.3. Practical implications

For user entrepreneurial enterprises aiming to enhance their performance, it is crucial to fully leverage the entrepreneurial ecosystem within the makerspace, embedding themselves at both structural and relational levels. First, user entrepreneurial enterprises should capitalize on the structural embeddedness of the makerspace, actively engaging in resource sharing, collaboration opportunities, and innovation environments. By immersing themselves in the makerspace’s ecosystem, they can access a wealth of support, resources, and tools. Networking with fellow entrepreneurs, investors, and professional mentors can provide invaluable feedback, guidance, and expertise, thereby accelerating product development, enhancing marketing strategies, and ultimately improving overall entrepreneurial performance. Second, user entrepreneurial enterprises must prioritize the development of strong relational embeddedness within the makerspace. Building solid cooperative relationships and social networks is essential for fostering information exchange, facilitating technology transfer, and stimulating innovative thinking. Through cultivating these relationships, enterprises can benefit from cross-border collaborations and unlock new business opportunities. Establishing mutually beneficial connections with other entrepreneurial teams or companies along the relevant industry chain can enable enterprises to collectively address challenges, explore new markets, and strengthen their competitiveness and long-term sustainability. In doing so, user entrepreneurial enterprises can create a robust foundation for growth and success within the dynamic makerspace environment.

Makerspaces encounter several challenges in fostering network embeddedness for user entrepreneurial enterprises, such as the difficulty of quantifying embeddedness, inconsistencies in policy implementation, and variations in entrepreneurs’ willingness to engage in collaborative networks. Addressing these issues is crucial to ensuring that network embeddedness is effectively leveraged to enhance user entrepreneurial success. To optimize embeddedness, systematic evaluation methods, such as Social Network Analysis (SNA), can be adopted by makerspaces, with key indicators like network centrality (a firm’s position within the network), tie strength (the frequency and trust in interactions), and information flow (the efficiency of resource exchange) used to assess the enterprises ‘ embeddedness. These assessments help refine network structures and improve the efficiency of resource allocation. Furthermore, industry networking events, entrepreneurial mentorship programs, and interdisciplinary collaborations should be implemented to strengthen connections, foster knowledge sharing, and promote resource integration among firms. Additionally, structured entrepreneurial support services, including mentorship, technical assistance, and market expansion strategies, can accelerate firm growth. At the same time, the cultivation of an open and collaborative entrepreneurial culture is essential, with entrepreneurs being encouraged to actively engage in network interactions and co-creation processes. By implementing these strategies, the benefits of network embeddedness can be maximized, creating a dynamic innovation ecosystem that supports the long-term development of user entrepreneurial enterprises.

To address these challenges, government departments should increase funding and policy support to strengthen the social networks of makerspaces. This can be achieved by providing financial support and tax incentives, such as entrepreneurship subsidies, tax reductions, or fiscal support for makerspace infrastructure, which would enhance service quality and innovation. Additionally, incentive policies, including tax breaks, preferential loans, or equity investments, should be introduced to encourage enterprise and investment participation, thus attracting more resources and capital to expand makerspaces’ social networks. Makerspace management can also be regulated through laws and regulations to ensure efficient resource allocation and foster cross-industry collaboration, with operational standards and resource-sharing mechanisms promoting inter-sectoral cooperation. Furthermore, collaboration with universities and research institutions could provide technology transfer channels and entrepreneurial training programs, offering essential technical and talent support. In addition to financial and technical support, long-term cooperation mechanisms between the government and makerspaces should be established. By organizing activities like entrepreneurship competitions, innovation forums, and networking events, the government can foster closer cooperation among makerspaces, creating a more integrated and synergistic business network. These measures will enhance makerspaces’ operational effectiveness and contribute to the sustainable development of user entrepreneurial enterprises.

### 6.4. Limitations and future research

This study has several limitations. First, while it primarily examines the impact mechanisms of structural and relational embeddedness in makerspaces on user entrepreneurial performance, the role of cognitive embeddedness should not be overlooked. Future research should adopt a more comprehensive approach by considering all three dimensions of network embeddedness or their combinations to gain deeper insights into their effects on user entrepreneurial performance. Additionally, further exploration is needed regarding how firm size and industry type moderate these relationships. For instance, larger firms, benefiting from greater resource availability, may derive more advantages from network embeddedness, whereas smaller firms, due to their flexibility, may respond more effectively to network dynamics. Similarly, industry type may shape the effects of embeddedness, with firms in high-tech industries relying more on strong ties for critical technological support, while those in traditional industries may benefit more from weak ties that provide broader market information. Future studies could develop specific theoretical hypotheses based on these moderating effects and employ appropriate empirical methods to further examine the underlying mechanisms.

Second, due to constraints in time and resources, the sample selection in this study is relatively limited, potentially leading to regional and cultural biases, particularly concerning the geographical scope and the cross-sectional nature of the data. To enhance the generalizability and applicability of the findings, future research should expand sample coverage, increase regional and industry diversity, examine longitudinal effects, explore the impact of technological advancements, and conduct cross-national comparative studies across different business networks and cultural contexts.

Finally, this study uses hierarchical regression analysis to test direct effects among the proposed hypotheses (Oo et al., 2019) and uses the “PROCESS” macro with the bootstrap method (Preacher and Hayes, 2008) to assess mediation effects. This methodological combination ensures model robustness while clearly delineating the pathways of variable interactions. However, future research could adopt structural equation modeling (SEM), which allows for the simultaneous analysis of complex relationships among latent constructs, including direct, indirect, and mediating effects—an approach well aligned with this study’s theoretical framework. Through SEM, future studies can provide a more comprehensive and in-depth understanding of the interplay between network embeddedness, business model innovation, and entrepreneurial performance.

## Supporting information

S1 Data Showing: pone.0322388.s001.xlsx (XLSX)
